# Microbes as Medicines: Harnessing the Power of Bacteria in Advancing Cancer Treatment

**DOI:** 10.3390/ijms21207575

**Published:** 2020-10-14

**Authors:** Shruti S. Sawant, Suyash M. Patil, Vivek Gupta, Nitesh K. Kunda

**Affiliations:** Department of Pharmaceutical Sciences, College of Pharmacy and Health Sciences, St. John’s University, Jamaica, NY 11439, USA; shruti.sawant19@my.stjohns.edu (S.S.S.); suyash.patil19@my.stjohns.edu (S.M.P.); guptav@stjohns.edu (V.G.)

**Keywords:** bacteriotherapy, cancer therapy, tumor targeting, bacterial vectors, bacterial tumor immunotherapy, prodrug therapy, quorum sensing

## Abstract

Conventional anti-cancer therapy involves the use of chemical chemotherapeutics and radiation and are often non-specific in action. The development of drug resistance and the inability of the drug to penetrate the tumor cells has been a major pitfall in current treatment. This has led to the investigation of alternative anti-tumor therapeutics possessing greater specificity and efficacy. There is a significant interest in exploring the use of microbes as potential anti-cancer medicines. The inherent tropism of the bacteria for hypoxic tumor environment and its ability to be genetically engineered as a vector for gene and drug therapy has led to the development of bacteria as a potential weapon against cancer. In this review, we will introduce bacterial anti-cancer therapy with an emphasis on the various mechanisms involved in tumor targeting and tumor suppression. The bacteriotherapy approaches in conjunction with the conventional cancer therapy can be effective in designing novel cancer therapies. We focus on the current progress achieved in bacterial cancer therapies that show potential in advancing existing cancer treatment options and help attain positive clinical outcomes with minimal systemic side-effects.

## 1. Introduction

Conventional anti-cancer therapy generally involves the use of surgery, chemical chemotherapeutic agents, and radiation therapy. However, a major problem when using conventional anti-cancer therapeutics is non-specific toxicity to normal cells and poor penetration into tumor cells. Moreover, prolonged usage of chemotherapeutics has led to the development of drug resistance resulting in ineffective treatment. This has served as a driving force for the development of alternative anti-tumor therapeutics [1]. Vaccines, in the form of attenuated bacteria and viruses, have been used to provide immunity against pathogenic infections and are now being investigated for anti-tumor properties to widen the scope of novel anti-cancer therapeutics.

The advent of microbial therapy for cancer treatment was first reported in 1867, by a German physician Dr. Willhem Busch, who highlighted a case of cancer remission when the patient contracted erysipelas (now known as *Streptococcus pyogenes*) [2]. In the late 19th century, Dr. William B. Coley, a surgeon at the New York Hospital observed regression in tumors after the patients developed skin infection due to Streptococcal bacteria. Dr. Coley posited the hypothesis that these infections stimulated the immune system to curb the tumor growth. He then started injecting the patients with live cultures of *Streptococcus* to inspect the tumor regression, however, some fatalities were observed. In response to this, Coley administered mixture of lysates obtained from *Streptococcus pyogenes* and heat-killed *Serratia marcescens*, commonly termed as Coley’s toxin which is considered as the first example of cancer immunotherapy [3,4]. The serendipitous benefit of concurrent infection due to the presence of pathogenic bacteria in tumors led to the investigation of bacterial activity against cancer. 

The motility of bacteria is responsible for swimming in the vasculature and effective penetration into tumors. Bacterial infections activate the innate and adaptive immune system which not only targets the bacteria but also, tumor cells. This ability of the bacteria has been used to develop bacterial anti-cancer therapy. An essential step in the development of bacterial therapeutics is the identification of potential species and strains of bacteria possessing minimal pathogenicity to the host [5]. An ideal anti-cancer bacterium should be: (i) non-toxic and non-immunogenic to the host, (ii) should replicate only in the tumor, (iii) spread evenly throughout it, and (iv) cause lysis of the tumor cells [6]. The hypoxic environment of the tumor tissue is suitable for colonization and supports the growth of bacteria (known as tissue tropism). For instance, anaerobic bacteria such as *Clostridia* species, have the tendency to preferentially accumulate and grow in tumors rather than normal cells due to the conducive tumor environment. This favors anaerobic growth of bacteria and offers protection from the host immune system [7]. In addition, competition for essential nutrients between bacteria and tumor cells may also result in anti-tumor effect. 

In this review, we aim to introduce and summarize bacterial-based anti-cancer approaches. Genetic engineering and synthetic biology are essential tools to bioengineer therapeutic bacteria to maximize their efficiency in cancer treatment. Bacteria can be genetically engineered and used as direct oncolytic agents or as vectors for delivery of gene therapy, cytotoxic agents, and peptides [8,9,10]. Species of *Clostridia, Bifidobacteria*, and *Salmonellae* are commonly used as vectors for delivery of tumor suppressor genes, tumor associated antigens, or suicide genes [8]. The use of bacterial anti-cancer therapy in conjunction with the conventional cancer therapy as effective treatment options have also been described.

## 2. Mechanism of Tumor Suppression 

**Figure 1:**
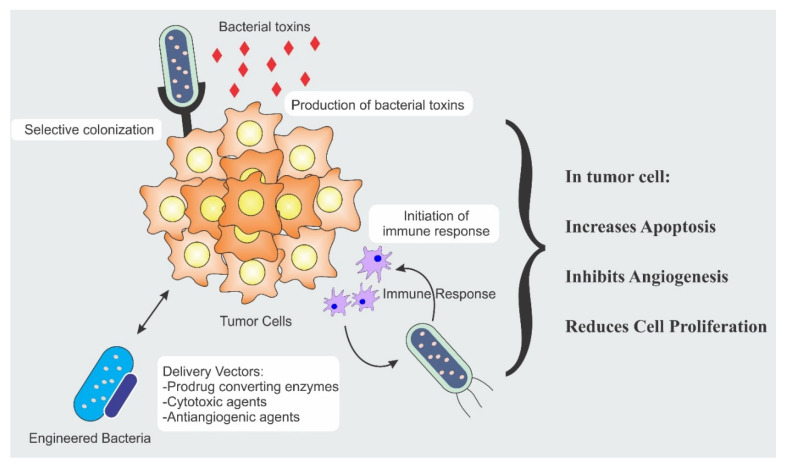
Bacterial therapeutic mechanisms involved in tumor eradication.

The bacterial anti-cancer therapy is an important weapon in the arsenal of fight against cancer. Strength of the bacterial therapy lies in its specific targeting to the cancer cells. Targeting is achieved by different modalities and each modality has a distinct mechanism. Different mechanisms responsible for anti-cancer activity include secretion of cytotoxic agents, immune engagement by the bacteria, and engineered bacterial vectors for the expression and release of tumoricidal proteins [11,12,13]. Figure 1 illustrates the different mechanisms involved in tumor suppression. 

### 2.1. Bacterial Cell Death Inducing Agents

A wide range of bacterially derived products, discussed below, such as cytotoxic factors/bacterial toxins, enzymes, peptides, and other secondary metabolites can specifically target the cancer cells and exert a cytotoxic effect [14]. Table 1 summarizes the therapeutic potential of these agents along with their anti-cancer mechanisms.

#### 2.1.1. Bacterial Toxins

Bacteria express and release certain toxins that inhibit the production and release of antibodies and cytokines resulting in immunosuppression. Bacterial toxins are enzymatic in nature and have a high degree of specificity for cellular substrates which are signaling molecules. The toxins may be cell cycle inhibitors, mitosis blockers and may compromise immunity by preventing lymphocyte clonal expansion [15]. The bacteria on entering the tumor cells release the toxins and alter their substrates, change the functions and morphology of the cells, and eventually kill its host. Bacterial toxins can be cytotoxic or at lower concentration may result in alteration in proliferation, differentiation, and apoptosis. These alterations can cause cellular aberrations and result in carcinogenesis [16].

The bacterial toxin widely used as an anti-tumor agent is cytolysin (CylA). Cytolysins are pore forming agents, that form multimeric pores in the eukaryotic membrane and induce apoptosis by the caspase-mediated pathway. Cytolysins are generally derived from *Escherichia coli* or *Staphylococcus aureus* [4]. Studies have demonstrated that mice on treating with *S. typhimurium* or *E. coli* strains expressing the ClyA toxin demonstrated tumor growth inhibition [11,17]. Jiang et al. engineered cytolysin producing *E. coli* strain K-12 which in combination with radiotherapy retarded tumor growth in mice with tumors derived from murine CT26 colon cancer and prevented its metastatic spread [11]. The bacteria *Clostridium botulinum* produces Botulinum neurotoxin A, which diminishes cell proliferation in prostate cancer (PC-3 and LNCaP) [18] and induces apoptosis in breast cancer cell lines (T47D) [19,20]. In another study by Lim et al., modified Pseudomonas exotoxin A (PE38), a natural ligand of epidermal growth factor receptor (EGFR) when expressed in *Salmonella typhimurium*, led to the inhibition of the solid tumor growth in mice implanted with colon cancer cells and 4T1 breast cancer cells having considerable EGFR expression [21].

#### 2.1.2. Bacterial Enzymes

Essential amino acids are vital for normal cell growth and cellular metabolism. These amino acids can serve as a limiting factor, capable of regulating the uncontrolled, rapid growth of tumor cells. Deprivation of these essential amino acids is an alternative therapy for cancer. To do so, bacteria produce a wide array of enzymes that act on these essential amino acids and prevent various cellular activities required for tumor growth. L-Asparaginase, arginine deiminase, and arginine decarboxylase are the commonly investigated bacterial enzymes [22]. L-Asparaginase (L-ASNase) catalyzes the hydrolysis of asparagine, enzymes such as arginine deiminase and arginine decarboxylase are involved in arginine catabolism. These enzymes result in deprivation of the amino acids required in protein synthesis and subsequently cause tumor cell death [23].

*Escherichia coli*, *Erwinia* species, *Streptomyces*, and *Bacillus subtilis* species are the sources of L-ASNase. In a study by Nguyen et al., L-ASNase enzyme caused lowering of blood asparagine levels thereby inhibiting protein synthesis and causing cell cycle arrest in G1 phase of leukemia cells [24]. L-ASNase has been proved effective in the treatment of acute lymphoblastic leukemia, lymphosarcoma, neoplasia, and other malignancies [24,25]. Commercial asparaginase have L-glutaminase activity, are antigenic, and cause hypersensitivity reactions. The L-glutaminase coactivity is responsible for the side effects such as hepatotoxicity and immunosuppressive effect. It has been reported by Nguyen et al. that *Erwinia chrysanthemi* derived L-ASNase is highly effective in the treatment of acute lymphoblastic leukemia demonstrating lower side effects and is less toxic as compared to the other FDA approved asparaginases as it lacks L-glutaminase coactivity [24].

#### 2.1.3. Bacteriocins

Bacteria produce a broad spectrum of ribosomal synthesized bacteriocins and antimicrobial peptides (AMP) which prevent the invasion of other bacterial strains in its own niche. The bacterial peptides can also inhibit the growth of tumor cells. Bacteriocins have an anti-cancer effect due to the cationic charge and amphiphilic nature [38]. This characteristic enables the bacteriocin to act as “membrane active” by interacting with the negatively charged cell membrane. The electrostatic interaction between cationic bacteriocins and cancer cells play an important role in cytotoxic effect. The cancer cells also have low cholesterol levels and with the cancer progression, the membrane fluidity increases making the cancer cells more susceptible to the action of AMPs [38]. Bacteriocins can destroy the membrane integrity and trigger the apoptosis of the cancer cells. Other mechanism involves the inhibition of angiogenesis and hence, impeding progression of cancer [39]. For instance, Nisin—a 34 amino acid residue produced by *Lactococcus lactis* subspecies *lactis* has antimicrobial activity against majority of the Gram-negative bacteria and has also demonstrated to be cytotoxic to MCF-7 cells (human breast adenocarcinoma cell line) [40]. *Brevibacillus* species produce defensin like peptide Laterosporulin 10 which was found to be cytotoxic against cancer cell lines like MCF-7 (human breast adenocarcinoma cell line), HeLa cells (uterine cervix cancer cells), HEK293T cells (human embryonic kidney), and HT1080 (fibrosarcoma) cell line [41]. 

#### 2.1.4. Biosurfactants

The extracellular, amphiphilic surface-active compounds produced by microbes are referred to as biosurfactants. An interesting utility of these biosurfactants as anti-cancer agents is noteworthy. Biosurfactants interfere with the cancer progression processes by inhibiting crucial signaling pathways such as PI3K/Akt, JAK/STAT pathway and MAPK signaling. In addition, biosurfactants can stimulate Natural killer (NK) cells, inhibit angiogenesis, and induce apoptosis via death receptors in cancer cells [42,43]. Surfactin, a cyclic lipopeptide biosurfactant has demonstrated anti-cancer activity by cell cycle arrest, apoptosis, and inhibiting metastatic escape. The ability of these surfactin molecules to be incorporated in nanoformulations such as polymeric nanoparticles, micelles, and liposomes is a major advantage in targeting the cancer cells for improved delivery [44]. Surfactin from Bacillus safensis F4 exhibited significant anti-biofilm and anti-tumoral efficacy against T47D breast cancer cells and B16F10 mouse melanoma cells [45]. Biosurfactant isolated from the bacteria Acinetobacter indicus M6 demonstrated anti-proliferative activity in lung cancer cell line A549 and the non-toxic nature of biosurfactants was demonstrated on the non-tumor MC-3 T3-E1 cell line. The G1 phase was arrested in A549 cells indicating inhibition of DNA synthesis as the mechanism of cell death. The protein moiety of the biosurfactant was responsible for the lysis of the cell membrane, owing to its detergent activity [46]. Rakicidins from marine bacterium *Micromonospora*, possesses hypoxia selective toxicity against several cell lines such as colon cancer (HCT-8), lung cancer cells (A549), gastric cancer (SGC7901), uterine cervix cancer cells (HeLa), and hepatocellular carcinoma cells (HepG2). Rakicidins induced apoptosis by activation of caspase-mediated pathways and by blocking signaling pathways such as MAPK and JNK/p38 [42].

### 2.2. Genetically Engineered Bacteria for Anti-Cancer Therapy

**Figure 2:**
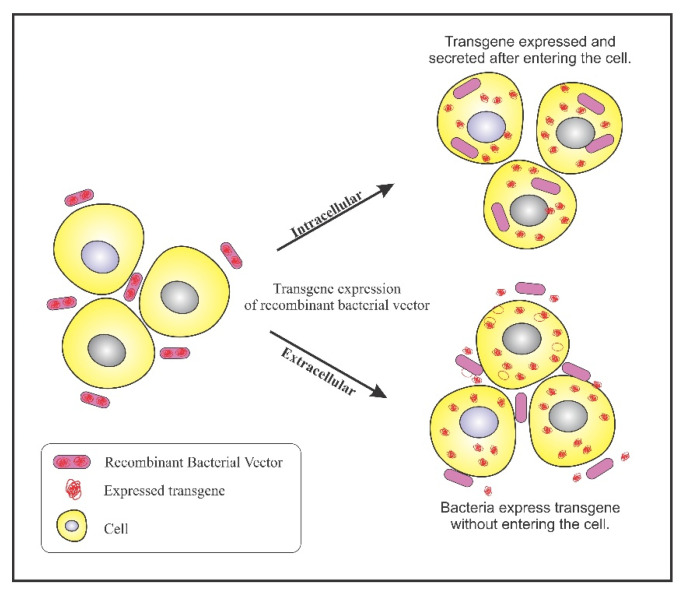
Bactofection into tumor cells: Intercellular: Bacteria bearing the transgene penetrates into the tumor cells, undergo lysis and release the transgene inside the tumor cell; Extracellular: Bacteria bearing the transgene do not enter the cell but, expresses the transgene in the vicinity of the tumor cells.

Bacterial ability to colonize in tumors has been exploited to design genetically engineered bacteria for confining its activity to tumor sites. A plethora of genetically engineered bacteria have been generated depending on their specificity of accumulation. A multitude of reporter genes, cytotoxic proteins, and tumor specific antigens can be expressed using genetically engineered bacteria as a vector. The engineered bacterium enters the target cells and expresses the transgene, resulting in the expression of therapeutic proteins. This process is referred to as bactofection. The invasive bacteria can deliver genes intracellularly into the tumor cells thus, fostering bactofection into tumor cells whereas the non-invasive strains are engineered to release therapeutic proteins external to the tumor microenvironment (TME) [47]. Figure 2 depicts the process of bactofection of engineered bacteria in tumor cells. Bioengineering bacteria for carrying the therapeutic cargo involves critical steps like virulence attenuation, enhancement of tumor targeting, and strategies for delivery of the anti-cancer cargo.

#### 2.2.1. Virulence Attenuation

Pathogenicity of bacteria is a major safety concern when employing them as vectors. Attenuation of pathogenicity and safety can be ensured by deleting certain bacterial virulence genes without affecting the anti-tumor activity. Liposaccharide (LPS) present in the outer membrane of Gram-negative bacteria is responsible for sepsis as it is a potent tumor necrosis factor-α (TNF-α) stimulator. Therefore, when using Gram-negative bacteria, various strategies are employed to address LPS-related complications. For example, safety profile of genus *Salmonella* was raised by deleting the virulence gene mshB which leads to loss of myristoylation of lipid A, an essential component of LPS. Hence, the toxicity of *Salmonella* was drastically reduced by minimizing the TNF-α expression [48]. This marked the generation of an attenuated *S. typhimurium* strain, VNP20009, by deleting msbB (lipid mutataion) and purI (purine auxotrophy) gene. The attenuated strain showed favorable tumor specificity and inhibitory action in various murine tumor models such as B16F10 melanoma and Lewis lung carcinoma [49]. However, in Phase I clinical trials, the strain lacked tumor specificity in metastatic melanoma patients. Tumor colonization was observed however, no anti-tumor effect was evident [50]. The failure was attributed to the presence of penta-acylated lipid A, an antagonist of toll-like receptor (TLR4) responsible for diminished immunostimulatory activity [51]. To address the limitation and introduce anti-tumor activity, mutant *Salmonella* strains were generated by deleting pagP, pagL, and lpxR genes thus forming hexa-acetylated lipid A possessing high affinity for TLR4. The anti-tumor activity was introduced with loss of virulence [1]. Another strain was genetically designed to downregulate genes responsible for expression of endotoxin associated genes. *Salmonella* relA- and spoT-mutant strains were altered causing malfunctioning in the synthesis of ppGpp, a signaling molecule involved in expression of toxic genes and thereby, improving the safety [2,52]. Its excellent anti-tumor activity can be attributed to inflammasome release such as nucleotide binding domain, leucine rich containing, pyrin domain containing (NLRP3) and ice protease activating factor (IPAF, also referred to as NLRC4) which induces the expression of proinflammatory cytokines, interleukins (IL-1β, IL-18) and TNF-α and consequently, causes tumor regression. This strain is defective in entering and replicating into the host cells, and hence acts as an extracellular bacterium [12].

Tumor specific proliferation and virulence attenuation can also be attained by developing auxotrophic mutants with specific nutrient requirement found preferentially only in the TME. Salmonella A1-R strain is auxotrophic for leucine and arginine and preferentially colonizes in tumors enriched with these nutrients. The A1-R strain was found to be effective in various mouse models of cancers such as prostate [53], pancreatic [54], and ovarian cancer [55]. *S. typhimurium* strain, YB1, was engineered from SL7207 by incorporating essential gene asd in the hypoxia induced promoter region. This enables the strain to selectively survive in hypoxic TME and hence, reduces the toxicity to the normal tissues. Moreover, the deletion of purI and purD genes gave rise to the need of exogenous adenine, which increased the bacteria’s susceptibility to proliferate in purine rich tumor regions in CT26 colon carcinoma mice model [56]. 

#### 2.2.2. Tumor Targeting Enhancement

Facultative anaerobic bacteria such as *Salmonella* and *Listeria* can survive in the oxygenated environment and cause toxicity to normal tissues. Thus, enhancing the tumor targeting of facultative anaerobes is of prime importance. Two approaches can be implemented to potentiate tumor targeting—first, by genetically modifying the bacteria to display tumor specific ligands on its surface. For example, the α_v_β_3_ integrin is overexpressed on cancer cells and a *Salmonella* ppGpp-deficient strain SHJ2037 was designed to display integrin binding peptide Arg-Gly-Asp (RGD) onto the bacterial surface to target the cancer cells. The SHJ2037 strain demonstrated high tumor specificity and enhanced anti-tumor activity in MDA-MB-231 breast cancer cells and MDA-MB-435 melanoma xenografts with overexpressed α_v_β_3_ integrin [57]. The second approach is by genetically engineering bacteria to target tumor associated antigens. For this purpose, *Salmonella* strains with surface display of lymphoma associated antigen CD20 were engineered to carry prodrug converting enzyme herpes simplex virus thymidine kinase (HSV-TK). HSV-TK when co-administered with ganciclovir effectively treated mice with human lymphoma xenografts [58]. 

#### 2.2.3. Bacterial Vectors for Anti-Cancer Cargo

Bacteria serve as vehicles for a wide array of therapeutic payload in the form of DNA, RNA, or protein to be delivered to the tumor cells. The intracellular bacteria are engineered to mediate a plasmid-based gene, carrying gene expression cassettes that regulate the expression of therapeutic proteins inside the tumor [47,48]. Induction of apoptosis in tumor cells using ligands such as TNF-α, Fas ligand (FasL), TNF–related apoptosis inducing ligands (TRAIL) is an interesting therapeutic approach; however, toxicity and short circulation half-life after systemic administration limits this approach. This limitation can be addressed by using engineered bacterial strains that can help home apoptosis inducing ligands effectively into tumor cells [48]. Various studies have confirmed the anti-cancer potential of engineered bacteria expressing death inducers [10,59,60]. *S. typhimurium* was successfully engineered to secrete murine FasL, a pro-apoptotic cytokine, and demonstrated reduced tumor growth in murine breast carcinoma and CT-26 colon carcinoma cells [61]. In another study, *S. typhimurium* was designed to secrete TRAIL which induced tumor growth suppression in mice bearing melanoma tumor [10]. In both the cases, tumor growth was suppressed by induction of caspase-3 mediated apoptosis. Table 2 summarizes the applications of genetically engineered bacteria as a vector for anti-cancer treatment.

#### 2.2.4. Drug Expression Strategies

Bacteria can sense and respond to a variety of stimuli. This property is often exploited for production of anti-cancer entities such as genes encoding for immune modulating agents and enzymes specifically at the tumor site [62]. 

The control on gene expression is critical for delivery of therapeutic payload at the tumor site, especially to ensure that the remaining healthy cells are unaffected. Specificity can be achieved by inserting therapeutic agents under the control of promoters which are induced by factors such as hypoxia or radiation. Further, the gene expression can be regulated by externally inducible promoters (like chemical compounds –L-arabinose) or microenvironment sensing promoters (like γ-irradiation) [62]. Bacteria reside in close communities and communicate internally using signaling molecules. This mode of communication is referred to as quorum sensing (QS). The regulation of gene expression can also be controlled by these QS molecules [72]. The application of QS phenomenon in cancer treatment has been discussed in detail in Section 2.6.

Ryan et al. employed the fumarate and nitrate reduction (FNR) regulator in *Salmonella typhimurium* for expression of gene encoding for cytolysin. The FNR dependent promoter is highly sensitive to oxygen and activates gene expression under anaerobic conditions hence, making it suitable for hypoxic tumor regions. The *S. typhimurium* equipped with FNR dependent promoter demonstrated successful expression of the genes encoding for cytolysins in mammary tumors and resulted in reduced tumor growth [73].

Light inducible promoters are used for spatiotemporal modulation of gene expression. Activation of these light promoters occurs at different wavelengths and its use depends on tissue penetration and safety in the body. For instance, shorter wavelengths cannot penetrate the tissue however, near infrared light and red light can penetrate the deeper tissue parts. Magaraci et al. engineered *E. coli* to secrete cytolysin under the influence of pDawn, plasmid light responsive plasmid (optogenetic) and exhibited mammalian cell cytotoxicity [13].

Bacterial enzyme L-ASNase, has the capacity of inducing apoptosis by catalyzing inhibition of global protein synthesis. Kim et al. conducted studies to potentiate the performance of *Salmonella*. L-ASNase was selectively expressed within solid tumors under the control of quorum sensing system. In these QS systems, auto inducer molecules such as acyl-homoserine lactone are secreted based on the local cell density during bacterial growth. After reaching a threshold concentration, the Vibrio QS expression system is activated and hence, genes encoding for L-ASNase are expressed [74]. To test the tumor specific expression of proteins using the QS systems, quorum sensing lux system was cloned in non-pathogenic *Salmonella* with green fluorescence protein reporter gene. The protein expression was evidently seen only in the tumor tissues and not in healthy tissues and no expression was seen in case where the distance between the bacterial colonies was greater than 155 mm [75].

### 2.3. Bacterial Directed Enzyme Prodrug Therapy (BDEPT)

The BDEPT approach involves engineering bacteria to carry the gene encoding for a prodrug converting enzyme. The gene directed enzyme prodrug therapy involves two steps. In the first step, the engineered bacterial vector is delivered into the tumor. The bacteria then preferentially proliferate in the hypoxic and necrotic tumor where the enzyme is expressed. In the second step, the prodrug is administered systemically and upon accumulation at the tumor site, the expressed enzyme catalyzes the conversion of the prodrug into its active cytotoxic form. The in situ generation of the active drug at the tumor site promotes tumor selective cytotoxicity and reduces the chances of systemic toxicity [47,76].

In the BDPET approach, the enzyme expressing bacteria stimulate the killing of neighboring tumor cells that do not express the enzyme (known as bystander effect). The active metabolite of the prodrug can be transported to the neighboring tumor cells by passive diffusion or can be transported through gap junctions [77]. Thus, the colony of the bacterial cells in tumor stroma can cause tumor regression without invading into the cancer cells. The design of the BDEPT system is critical and factors such as enzyme orientations, enzyme kinetics, toxicity of the prodrug/active pharmaceutical ingredient to the bacterium, and pharmacokinetic properties of the drug should be considered. Selection of an appropriate bacterial carrier is vital considering the level of invasiveness—intracellular or extracellular, wild type or recombinant carrier, and nature of the bacterium—facultative or obligate anaerobic bacteria [78].

Several prodrug/enzyme systems have been investigated, with commonly employed activating enzymes being cytosine deaminase (CD), nitroreductase, thymidine kinase, and cytochrome P450 enzymes. The CD enzyme catalyzes deamination of the prodrug, 5-fluorocytosine (5-FC) into 5-fluorouracil (5-FU) which interferes with DNA and RNA synthesis. CD was expressed in bacterial species, *Bifidobacterium longum* and Clostridial species—C. *beijerinckii*, *C. acetobutylicum,* and *C. sporogenes* [79]. In an ongoing Phase I-II study (NCT01562626), 5-FC in conjunction with recombinant *Bifidobacterium longum* (APS001F) modified to produce CD is being evaluated for its efficacy and safety to treat solid tumors [80]. In another study by Nemani et al., *E. coli* was engineered to express CD at elevated temperatures [9]. The engineered bacteria were encapsulated in magnetic nanoparticles and expression was controlled by alternating magnetic field-induced hyperthermia. Combination of this system with 5-FC demonstrated cytotoxicity on glioma cells, prostate cancer, and breast cancer cell lines [9]. Afkhami -Poostchi et al. investigated the therapeutic efficacy of BDEPT by genetically engineering *E. coli* to carry DH5α-lux/βG plasmid containing β-glucuronidase enzyme encoding gene. This gene helped in the activation of natural, non-toxic product glycyrrhizic acid to active glycyrrhetinic acid in TME of colon carcinoma mouse model. Results were indicative of effective bacterial tumor colonization and proliferation in TME as observed from the fluorescence emitted by the bacteria. The combined treatment greatly increased apoptosis and reduced the tumor growth [81].

*Escherichia coli* Nissle 1917 was reprogrammed by Ho et al. to promote its binding to heparan sulfate proteoglycan on colorectal cancer cells and secrete the enzyme myrosinase. Myrosinase is responsible for conversion of glucosinolates present in cruciferous vegetables to sulphoraphane, a natural metabolite that inhibits tumor growth and promotes apoptosis in cancer cells. A reduced tumor regression and tumor occurrence was observed in murine models of colorectal carcinoma in comparison to the treatment of engineered bacteria or dietary glucosinolates alone [82]. To understand the importance of enzyme prodrug partnership for an effective therapy, Chan-Hyams et al. studied the bystander effect of various nitroaromatic prodrugs (metronidazole, CB1954, nitro-CBI-DEI, PR-104A, and SN27686) in combination with nitroreductase expressing *E. coli NfsA*. It was observed that reduced metabolites of metronidazole, PR-104A, and SN27686 exhibited little cell to cell transfer while nitro-CBI-DEI and reduced metabolites of CB1954 passed it effectively. It was also demonstrated that 2-nitro reduction products showed substantially higher bystander effect as compared to 4-nitro reduction products [83]. Lehouritis et al. exploited the natural enzymolome of the wild-type probiotic bacteria such as *Escherichia coli* Nissle, *Bifidobacterium breve*, *Lactococcus lactis*, and *Lactobacillus* species to activate multiple prodrugs owing to their safety. *E. coli* activated multiple drugs such as CB1954, 5-FC, AQ4N, and fludarabine phosphate simultaneously, and exhibited significant reduction in tumor growth in mice bearing subcutaneous CT26 murine colon carcinoma tumors. These findings demonstrated that wild strains of probiotic bacteria in conjunction with prodrug combinations were efficacious without the need for genetically engineering the bacteria [84].

### 2.4. Anti-Angiogenic Therapy

Solid tumors witness pronounced angiogenesis resulting in proliferation of vast network of blood vessels around the tumor cells. New blood vessels trigger the growth and metastasis of solid tumors. However, the newly formed blood vessels are highly disorganized and have anatomical differences in terms of incomplete endothelial linings. This results in leaky vasculature which allows residence of the circulating bacteria [85]. The combination of tumor targeting bacteria and inhibition of angiogenesis would thereby enhance the possibility of tumor eradication [86,87]. 

Shi et. al. engineered *Salmonella typhimurium* ST8 harboring a plasmid encoding for angiogenic inhibitor, Endostatin fused with Type III secretion system (T3SS) protein SopA. The T3SS proteins interfere with the pro-angiogenic action of the growth factors in tumors. Administration of ST8/pSEndo in murine CT26 colon cancer mice showed effective tumor regression with low blood vessel density and severe tumor necrosis as compared to a mock control ST8/pSGFP [8]. Potent anti-angiogenic and tumor vessel normalization properties of Histidine-proline-rich glycoprotein (HPRG) were introduced in *S. typhimurium* VNP20009 strain under the control of hypoxia-induced NirB promoter. VNP20009 mediated HPRG significantly reduced tumor growth and enhanced survival in primary B16F10 (melanoma mice model) and inhibited lung metastatic tumor growth in B16F10 metastatic tumor model [88]. Combination therapy of *S. typhimurium* VNP20009 with triptolide, an antiangiogenic and anti-inflammatory compound was investigated for melanoma treatment. Tumor targeting VNP20009 triggers the migration of neutrophils, macrophages, and CD8+ T cells. Macrophages and neutrophils have a bactericidal effect and may suppress VNP20009. However, the combination therapy with triptolide, reduced the infiltration of neutrophils in the tumor as well as reduced the expression of vascular endothelial growth factor (VEGF) resulting into larger necrosis in the melanoma [89]. 

Lipophilic AMPs from new endophytic bacterial strain, EML-CAP3 isolated from red pepper leaf (*Capsicum annuum* L.) were studied by Jung et al. The newly obtained AMP demonstrated potent tumor induced angiogenic inhibition by downregulating Hypoxia inducible factor-1 α and its target gene VEGF in human umbilical vein endothelial cells [90]. Guo et al. devised bacterial particles consisting of fixed bacteria *E. coli* (TOP10), double attenuated (aroA− and dam-) *Salmonella typhimurium* strain RE88, and *Streptococcus thermophilus.* The systemic administration of these bacterial particles in mice bearing subcutaneous CT26 colon cancer and LL2 Lewis lung cancer stimulated innate immune response by phagocytosis and inflammatory cytokines were released. The release of inflammatory cytokines enabled the bacterial particles to disrupt the tumor vasculature, induce tumor hemorrhage and necrosis resulting in extensive cell death in the TME. The effect of the destruction of the tumor vasculature due to the bacterial particles was similar to the combination of six inflammatory cytokines namely, TNF-α, KC, MCP-1, G-CSF, IP-10, and IL-6 [91].

Talib et al. investigated *Propionibacterium acnes* in combination with melatonin for breast cancer treatment in a mice model. The combination had a synergistic action in stimulating interferon gamma (INF-γ) production and inhibiting VEGF expression. The resulting effect was reduced angiogenesis, enhanced apoptosis, and strong Th1-type cytokine anti-tumor immune response that led to tumor regression [92]. Anti-angiogenic therapy comprising of gemcitabine/bevacizumab and subsequent administration of *Salmonella typhimurium* A1-R showed higher efficacy than the drug combination alone, in pancreatic cancer derived orthotopic xenograft model, hence indicating its potential in clinical applications [54].

### 2.5. Bacteria Tumor Immunotherapy

The immune system plays a critical role in tumor immunosurveillance. Abnormal cells are recognized and eliminated with the help of innate and adaptive immunity in a continuous and bidirectional manner [93]. Tumor evolution occurs in three phases of “immunoediting” namely—elimination, equilibrium, and escape phase. NK cells and cytotoxic T-Lymphocytes (CD8+) are the primary mediators in the process along with other mediators including macrophages associated to tumors, dendritic cells, naïve T cells, and regulatory T cells (T-regs). The microbial products and pathogen-associated molecular patterns lead to the activation of immune response [94]. Innate immunity is provided by the NK cells and these cells can recognize and eliminate the neoplastic cells. Once activated, NK cells accompanied by CD8+ T cells induce the recruitment and proliferation of other cells at the tumor site. This is attained by the release of cytokines such as interferon-gamma (IFN-γ), TNF, granulocyte and macrophage colony stimulating factor [1,93]. Cytokines induce apoptosis in tumor cells by anti-angiogenic effect on the tumor vasculature. For example, *Salmonella typhimurium* expressing and secreting recombinant IFN-γ demonstrated enhanced tumoricidal effect and prolonged the survival of melanoma tumor bearing syngeneic mouse model. The IFN-γ directly activated the NK cell and the macrophages leading to more effective tumor suppression [95]. Commercially available bioproduct of *Staphylococcus aureus* metabolites (consisting of enterotoxins, proteins, and amino acids) was found to be effective in the treatment of mesothelioma in orthotopic mouse models. The efficacy was attributed to the local activation of T-lymphocytes with significant activation of CD4+ and CD8+ T-cells leading to tumor necrosis [96]. Hydrodynamic tail vein injection of IL-21 in combination with attenuated *Salmonella typhimurium* (VNP20009), exhibited suppression of tumor growth and prolonged mice survival in a melanoma model. The smaller tumor size and longer survival time was observed in combination therapy than when administered alone. The anti-tumor immune response was attributed to the higher concentration of CD4+ and CD8+ lymphocytes and NK cells infiltration at the tumor site [97].

Bacteria also secrete certain immunomodulatory agents that are effective against cancer. Gram-negative bacteria produce outer membrane vesicles which induce anti-tumor responses by preferentially accumulating at the tumor site and stimulate the release of anti-tumor cytokines CXCL10 and IFN-γ [98]. To decipher the immune response pathways in bacteriolytic therapy employing *Clostridium novyi*—NT spores, immune cell function was analyzed in dogs developing neoplasia naturally. The study was aimed at studying the difference in immune response when the *Clostridial* spores were administered via the intravenous and intratumoral route. The intratumoral injection enhanced phagocytosis and NK cell like function however, intravenous injection exhibited increased lipopolysaccharide induced TNFα and lipoteichoic acid induced IL-10 production. A sustained increase in phagocytic and NK cell function indicated long term immune cell function changes which can possibly have association with tumor remission [99]. *Enterococcus hirae* and *Barnesiella intestinihominis* enhanced the immunomodulatory effects of cyclophosphamide by elevated CD8/T-reg ratio and promotion of interferon infiltration, respectively. The Th1 cell immune responses induced by these bacterial strains is predicted to have longer progression free survival in ovarian and lung cancer patients subjected to chemo-immunotherapy [100].

### 2.6. Bacteria Quorum Sensing for Tumor Targeting

Bacteria reside in close communities and establish cell to cell communication using signaling molecules. This mode of communication is referred to as quorum sensing (QS). The Gram-negative bacteria use N-acyl homoserine lactone whereas the Gram-positive bacteria use oligopeptides as signaling molecules. In addition, the autoinducer–2 synthesized by both the bacterial types is also used for signaling. Synthesis of these molecules occurs in a cell density dependent manner [101,102,103]. QS phenomenon can be used to design genetic circuits for identifying bacterial aggregation in tumor regions which can serve as biosensors [104]. Anderson et al. applied this technique to combine the genetic output interface encoding for invasin from *Yersinia pseudotuberculosis* in *E. coli* with the *lux* QS circuit of *Vibrio fischeri* having hypoxia-responsive (fdhF) promoter or arabinose inducible promoter (araBAD) to identify the tumor environment. Invasin enabled *E. coli* to invade into different cancer cells like epithelial, hepatocarcinoma, and osteosarcoma cell lines. The invasion mediated internalization is under the control of fdhF and araBAD promoters. This system can be used to engineer bacteria to sense the TME and propagate the entry and release of cytotoxic agents [105]. Chowdhury et. al. adopted the concept of quorum lysis in engineering *E. coli* strain for the local delivery of CD47 antagonist nanobody. The *E. coli* had a single plasmid encoding for synchronized lysis circuit, the bacteria expressing *luxI* proliferated and synthesized QS molecules such as acylhomoserine lactone. On reaching a critical threshold, bacteriophage lysis protein is produced resulting in release of the therapeutic cargo. The authors reported that the CD47 blockade in syngeneic mouse model boosted tumor infiltrating T cells activation leading to rapid tumor regression. A major advantage of this approach was induction of systemic anti-tumor immunity by local delivery of abundant adjuvants, which also precludes the generation of anti-drug antibodies and limits systemic toxicity [106,107]. 

The QS signaling molecules have also demonstrated anti-cancer potential. For instance, the D-Retroenantiomer of QS peptide (SYPGWSW) obtained from *Clostridium acetobutylicum* possess the ability to cross the blood brain barrier and has excellent tumor homing properties. PEGylated micelles were prepared having surface modification with QS peptide for intracranial glioma targeting. These surface modified micelles when loaded with paclitaxel exhibited excellent tumor accumulation and strong anti-angiogenic and anti-tumor effect in the glioma region [108]. The quorum sensing signal molecule, N-3-oxo-dodecanoyl-L-homoserine lactone (O-DDHSL) exhibited cytotoxic effect by inducing apoptosis, inhibiting colony formation, and reducing colony formation in pancreatic carcinoma cells [109]. O-DDHSL has also proved to mitigate proliferation and viability and induced significant necrosis in malignant MDA-MB-231 breast cancer cells [110]. Nandakumar et al. synthesized various QS analogs and tested its activity on Hodgkin’s lymphoma cells. The molecules proved to be cytotoxic and inactivated the NF-κB signaling in these cells [111]. 

Some microbiome related QS peptides from commensal or pathogenic bacteria such as the extracellular death factor (EDF) synthesized by the gut bacterium *E. coli*, PhrG peptide produced by *Bacillus subtilis*, a gut bacterium, and competence stimulating peptide from oral commensal bacteria *Streptococcus mitis* exhibit pro-invasive characteristics and angiogenic properties [103]. The EDF derived peptides and Phr0662 (*Bacillus* sp.) peptide from the human microbiome, have the possibility of causing cancer metastasis [102]. Thus, the identification of potential tumor targets and development of antagonists of such peptides can be a breakthrough in cancer therapy. A thorough exploration and understanding of the crosstalk between bacterial signaling molecules and TME is necessary to develop quorum sensing peptide agonists and antagonists for therapeutic effect in oncology. 

### 2.7. Biofilm Based Anti-Cancer Therapy

Bacteria form spatially structured, dense networks of colonies in extracellular polymeric matrix, adhering to biological or non-biological surfaces referred to as biofilms. The phenomenon of quorum sensing regulates the process of biofilm formation, which will further enable the bacteria to persist in the host cells and escape the host defense immune system [112]. In addition, the biofilm formation can result in the progression of colon and colorectal carcinogenesis [113]. Biofilm can be exploited as potential anti-cancer agents due to its ability to deliver therapeutics and to curb the spread of metastatic tumors.

Anti-cancer agents induced and promoted biofilm formation in *Pseudomonas aeruginosa* during cancer treatment with hydroxyurea and doxorubicin [114]. To escape the drug attack, the bacteria growing on the cancer cells are triggered by an SOS response, which is an inducible DNA damage repair system [115]. This leads to the development of various unique bacterial phenotypes that attack or penetrate the cancer cells. Also, DNA and many proteins are released by the bacteria which coat the cancer cells and halt the metastatic escape of the tumor [116,117]. Kumeria et al. fabricated naturally produced nanowires found in the form of biofilms of zetaproteo bacteria *Mariprofundus ferrooxydans* as a potential drug carrier for cancer therapy. Biofilm derived nanowires were found to be magnetic nanomaterial capable of generating active and passive trigger response with alternating magnetic field. The hyperthermia induced by the alternating magnetic field contributed to decreased cell viability. The biofilm derived nanowires were tested for their carrier capabilities by loading doxorubicin and were found to cause cytotoxicity in human breast cancer cells (MDA-MB231-TXSA) [118]. In another study, biofilm synthesized by *Lactobacillus reuteri* was used to formulate mesoporous silica nanoparticles for the targeted release of 5-FU in the colorectum and this biofilm when coated with zinc gallogermanate facilitated colorectal cancer imaging [119]. 

## 3. Conventional Cancer Therapy in Combination with Bacteriotherapy

The conventional modalities of cancer treatment such as chemotherapy, radiotherapy, and surgery have been used extensively. However, the chances of systemic toxicity, metastatic potential, development of secondary tumors, and possibilities of drug resistance may lead to ineffective treatments [120,121]. The chemotherapeutic agents encounter issues like poor tumor targeting, inadequate tumor penetration, and low cytotoxicity to cancer cells. Further, tumor hypoxia reduces the sensitivity of tumors to ionizing radiation and chemotherapeutics [122]. To counter these obstacles, integration of the conventional modalities of treatment with bacterial cancer therapy can introduce new approaches for achieving better clinical outcomes. 

Bacteriolytic *Salmonella* therapy in conjunction with cyclophosphamide decreased tumor vascularization and suppressed the tumor in melanoma model [123]. Bascuas et al. investigated the effect of *Salmonella* (LVR01 attenuated strain) in conjunction with CHOP (cyclophosphamide, doxorubicin, vincristine, and prednisone) chemotherapy in non-Hodgkin lymphoma bearing mice. Enhanced immune response was observed due to large influx of immune population, increased chemokines, proinflammatory cytokines, and activation of NK cell cytotoxicity. The combination of *Salmonella* immunotherapy demonstrated profound immune response and greater tumor activity than the CHOP therapy alone [124]. The *C. novyi*-NT spores in combination with microtubule interacting drugs such as vinorelbine and docetaxel demonstrated tumor regression in colon cancer xenograft models (HCT116). The combination therapy led to the destruction of both, the vascular and avascular components of the tumor [125]. In a recent study by Saltzman et al., the authors observed that immunotherapy provided by *Salmonella typhimurium* in combination with low dose doxorubicin, reduced the tumor burden in murine breast cancer model. The combination showed greater tumor reduction and lower toxicity than either therapy alone [126]. Zhang et al. devised paclitaxel liposomes and internalized them in bacteria (known as paclitaxel-in-liposome-in-bacteria, LPB) *E. coli* and *L. casei* for the treatment of lung cancer. The intratracheal administration of these bacterial formulations led to the enhanced immune cell migration (neutrophils and leucocytes) into the tumor microenvironment and the lung tumor tissues showed higher levels of TNF-α, IL-4, and IFN-γ than healthy lung tissues. The synergistic effect of the immunostimulation and high drug distribution of paclitaxel in the lungs resulted in successful anti-tumor effect [127].

To explore the combination of bacterio- and radiotherapy, *Salmonella* strain VNP20009 was coated with photothermal agent, polydopamine. The bacteria aids in the penetration of the photothermal agent to the core hypoxic region of the tumor and the biomimetic polydopamine on the surface of the bacteria aids in the conversion of near-infrared light into heat which causes tumor ablation in mice bearing malignant melanoma. The photothermal therapy kills tumor cells and generates cell lysates, which act as antigens to generate immune response and promotes bacterial growth. The tumors were eliminated and no relapse or metastasis was observed after one injection and irradiation [128,129]. Intratumoral administration of *Pseudomonas aeruginosa* in combination with localized low frequency square pulsed magnetic field exposure was effective in Ehrlich tumor treatment. Limited bacteria related toxicity and limited tissue injury contributed to the effectiveness of this combination [130]. Branched gold nanoparticles coated with *C. novyi* NT were fabricated and used with computed image tomography for delivery in prostate cancer mouse models [131]. The nanoparticle coated spores delivered to the hypoxic region were effective in treating the central tumor regions due to localization of the anaerobic *Clostridium* spores however, normoxic peripheral cancer cells remained alive. To prevent tumor recurrence, a nano biotherapeutic emulsion comprising of multifunctional nanoscintillators and *C. novyi* NT spores was formulated for the destruction of both the normoxic peripheral tumor and the central hypoxic tumor by image guided X-Ray photodynamic therapy [132]. The combined effect of bacterial prodrug therapy of *E. coli* nitroreductase/prodrug CB1954 and γ–rays was investigated for treatment of cervical carcinoma. This combination significantly enhanced the radio sensitivity of HeLa cells and had a marked effect on its cytotoxicity [133].

## 4. Challenges in Bacterial Cancer Therapy

Cancer is a multifactorial disease and the use of bacteria for cancer therapy as an immunostimulatory agent or as a vector for carrying the therapeutic cargo is a promising treatment method. Several clinical trials are underway to test the efficacy of bacterial therapy on human subjects (Table 3). However, a major problem with bacteria mediated cancer therapy is the toxicity. The dose for obtaining therapeutic effect may be toxic and have deleterious effects whereas lower doses can affect the treatment efficacy [15]. The balance between the benefit and safety of the trial subject must be maintained. Appropriate techniques and measures must be adopted to evaluate the immune response of the subject and the overall therapeutic benefit [134]. Differences in tumor structure between preclinical animal models and the human subjects can impact bacterial penetration and proliferation in the tumor [5]. Hence, optimization of the dose and route of administration is critical. Moreover, bacterial clearance by the immune system before reaching the tumor site may result in the failure of treatment [135]. Also, any mutations in the bacteria may result in the therapeutic loss and cause exaggerated infections. However, the use of recombinant DNA technology has mostly solved the safety concerns [15]. 

## 5. Conclusions

Cancer therapy has witnessed a paradigm shift with the advent of bacteriotherapy. The remarkable ability of bacteria to achieve target specificity and deliver a diverse range of therapeutic cargo marks the success of bacterial mediated cancer therapy. The tools of synthetic biology and genetic engineering have made it possible for tailoring bacteria to carry the therapeutic cargo efficiently. The combinatorial approaches of bacteriotherapy with chemotherapy and radiotherapy can help in defeating the tumor heterogeneity thus, resulting in positive outcomes. However, the challenges of safety and in vivo distribution of the bacteria remain a concern. Further investigations and developments in the field of bacterial therapy can provide a new dimension to cancer treatment.

## Figures and Tables

**Table 1:** Examples and anti-cancer mechanism of bacterial cell death inducing agents.

Bacterial Metabolites—Category	Bacterial Strain	Metabolite	Mechanism of Action	Biological Target: Cancer Cells/Cell Lines	References
Bacterial Toxins	*Corynebacterium diphtheria*	Diphtheria toxin (DT) 2 major fragments (DTA and DTB)	DTA is responsible for the cytotoxic enzymatic activity and inactivates the ADP-ribosylation of elongation factor 2; DTB facilitates cell entry by binding to surface receptors and subsequent translocation into cytoplasm by undergoing endocytosis	Ovarian cancer (SKOV-3), Pancreatic cancer (CRL-1687, CRL-2119, CRL-1997, and CRL-2547), Lung cancer (NCI-H460, NCI-H358, and A549)	[26,27,28]
	*Streptococcus*	Streptolysin O (SLO)	SLO binds to cholesterol in the plasma membrane, oligomerizes to form aggregates which form large pores. This results in cytolysis and cell death	Embryonic kidney fibroblasts (293T, HEK 293 cell derivatives that harbor SV40 large T antigen)	[29]
	*Listeria monocytogenes*	Listeriolysin O	Binds to cholesterol binding receptors and induces pore formation in the cell membrane resulting in cytolysis. Induces apoptosis in T-cells by caspase mediated pathway	Breast cancer cells (MDA-MB-231 and MCF-7)	[30]
	*Pseudomonas aeruginosa*	Exotoxin A	Targeting with tumor-related antigens and induction of cytotoxic pathways	Head and neck cancer cell line (KCCT873)	[31]
Bacterial Enzymes	*Mycoplasma hominis or M. arginine*	Arginine deiminase	Hydrolyzes arginine and deprives the tumor of arginine, essential for growth. This results in reduced tumor proliferation	Glioblastoma (HROG02, HROG05, HROG10)	[32]
	*Escherichia coli*	L-Asparaginase	Catalyzes asparagine hydrolysis and reduces its blood concentration. This results in selective growth inhibition of malignant cells	Breast carcinoma (MCF-7), Hepatocellular carcinoma (HepG2), Lung carcinoma (SK-LU-1)	[33]
Bacteriocins	*Lactococcus lactis*	Nisin A	Induces cell cycle arrest and apoptosis through activation of CHAC1	Colon cancer (SW480) cells	[34]
	*Escherichia coli*	Colicin	Binds to a specific receptor on the outer membrane and forms pores which leads to apoptosis	Lung cancer (H460, H292, and H23) cells	[35]
	*Staphylococcus bovis HC5*	Bovicin	Binds to the cell membrane and disrupts cell membrane integrity by pore formation. It also induces the potassium efflux in target cells	Breast cancer cells (MCF7), Liver cancer cells (HepG2)	[36]
Biosurfactants	*Bacillus subtilis*	Surfactin, a cyclic lipopeptide	Inhibits tumor cell invasion, migration, and colony formation	Breast carcinoma cells (MCF-7 and MDA-MB-231)	[37]

**Table 2:** Applications of genetically engineered bacteria as vectors for anti-cancer treatment.

Treatment Strategy	Bacterial Strain	Gene/Drug	Mechanism of Action	Application	References
Prodrug Therapy	*Salmonella*	Thymidine kinase polypeptide Prodrug: Ganciclovir	Inhibits deoxyguanosine triphosphate, dGTP, incorporation into DNA	Melanoma	[63]
Prodrug Therapy Anti-Angiogenic Therapy	*Escherichia Coli*	Uridine phosphorylase Prodrug: Capecitabine	Impede thymidylate synthase enzyme	Colon, rectum, and head and neck cancers	[64]
	*S. choleraesuis*	Endostatin	Increases infiltration of CD8(+) T cells	Melanoma, Bladder tumor, Hepatoma	[65]
Anti-Angiogenic Therapy Anti-Angiogenic Therapy Immunotherapy	*S. typhimurium SL7207*	VEGFR-2	Upregulates vascular-endothelial growth factor receptor 2 (FLK-1) of proliferating endothelial cells in the tumor vasculature	Melanoma, Colon carcinoma, Lung carcinoma,	[66]
	*S. typhimurium RE88*	IL-18	Activation of T, natural killer, and dendritic cells	Breast carcinoma	[67]
Anti-Angiogenic Therapy Immunotherapy Quorum Sensing peptides for anti-tumor action	*Serratia marcescens*	Endotoxin	Releases pro-inflammatory cytokines, making the immune system eliminate or protect against multiple tumors	Melanoma, Leukemia, Lymphoma	[68]
	*Listeria monocytogenes*	Listeriolysin O	Releases proinflammatory cytokines and increases expression of co-stimulant molecules in antigen presenting cells surfaces leading to maturation and activation of high affinity T cells	Prostate cancer	[69]
	*Pseudomonas. aeruginosa*	N-3-oxododecanoyl homoserine lactone (3OC12-HSL)	Inhibition by protein kinase	Cystic fibrosis	[70]
Biofilms as Anti-Cancer Agents	*Streptococcus agalactiae*	Polysaccharides	Inhibit adhesion of cancer cells to endothelial cells	Colon cancer	[71]

**Table 3:** Summary of the active clinical studies focused on bacterial mediated anti-cancer therapy [1,4,6].

Bacterial Strain	Gene/Strain	Tumor Model	Phase	Observation	Identifier (NCT Number)	Reference
*Salmonella* *typhimurium*	VNP20009 with HSV-TK	B16F10 melanomas	I	Dose-dependent suppression of tumor growth and prolonged survival	NCT00004988	[136]
	VNP20009	Metastatic melanoma, metastatic renal cell carcinoma	I	Induced a dose-related increase in the circulation of proinflammatory cytokines, such as IL-1β, TNF-α, IL-6, and IL-12	NCT00006254	[137]
	χ4550 with IL-2	Hepatoma, liver neoplasms	I	Consistent reduction in the mean number of hepatic metastases in fed animals	NCT01099631	[138]
*Salmonella typhimurium*	VXM01	Pancreatic cancer	I	Reduction in tumor perfusion after vaccination	NCT01486329	[139]
*Listeria* *monocytogenes*	JNJ-64041809	CT26 Colon tumor, Prostate cancer	I	Breaking of self-tolerance and long-term survival	NCT02625857	[140]
	GVAX+ CRS-207, Drug: Cyclophosphamide	Metastatic pancreatic cancer	II	Extended survival for patients with pancreatic cancer, with minimal toxicity	NCT01417000 NCT02004262	[141,142]
	ADXS11-001	Cervical Cancer	II, III	Promising safety and efficacy results	NCT02853604	[143,144]
*Clostridium*	*novyi-NT*	Solid tumor malignancies	I	Reduced the tumor size	NCT01924689	[145]
	*novyi-NT* *Drug: Pembrolizumab*	Refractory advanced solid tumors	I	Ongoing	NCT03435952	[146]

HSV-TK: Herpes simplex virus-1 thymidine kinase, IL: Interleukin, TNF: Tumor Necrosis Factor.

## Abbreviations

AMP	Anti-microbial peptides
BDEPT	Bacterial directed enzyme prodrug therapy
CD	Cytosine Deaminase
CD8+	Cytotoxic T-Lymphocytes
CHOP	Cyclophosphamide, doxorubicin, vincristine, and prednisone chemotherapy
ClyA	Cytolysin A
DT	Diphtheria Toxin
EDF	Extracellular death factor
EGFR	Epidermal growth factor receptor
FasL	Fas ligand
FNR	Fumarate and nitrate reduction regulator
HPRG	Histidine-proline-rich glycoprotein
HSV-TK	Herpes simplex virus thymidine kinase
IFN-γ	Interferon gamma
IPAF	Ice protease activating factor
L-ASNase	L-Asparaginase
LPS	Lipopolysaccharide
NK	Natural Killer cells
NLRP3	Nucleotide binding domain, leucine rich containing, pyrin domain
O-DDHSL	N-3-oxo-dodecanoyl-L-homoserine lactone
QS	Quorum sensing
RGD	Arg-Gly-Asp
SLO	Streptolysin O
T3SS	Endostatin Type III secretion system
TME	Tumor microenvironment
TNF–α	Tumor necrosis factor–α
TLR	Toll like receptor
TRAIL	TNF–related apoptosis inducing ligands
T-regs	Regulatory T cells
VEGF	Vascular endothelial growth factor
5-FC	5-Flurocytosine
5-FU	5-Fluorouracil
